# Dehydration-Driven Glass Formation in Aqueous Carbonates

**DOI:** 10.1021/acs.jpclett.5c00551

**Published:** 2025-05-07

**Authors:** Thilo Bissbort, Kai-Uwe Hess, Daniel Weidendorfer, Elena V. Sturm, Jürgen E. K. Schawe, Martin Wilding, Bettina Purgstaller, Katja E. Goetschl, Sebastian Sturm, Knut Müller-Caspary, Wolfgang Schmahl, Erika Griesshaber, Martin Dietzel, Donald B. Dingwell

**Affiliations:** † Earth and Environmental Sciences, Ludwig-Maximilians-Universität München, Theresienstraße 41/III, 80333 München, Germany; ‡ Laboratory of Metal Physics and Technology, Department of Materials, ETH Zurich, 8093 Zurich, Switzerland; § UK Catalysis Hub, Research Complex at Harwell, Rutherford Appleton Laboratory, Harwell Campus, Didcot, Oxfordshire OX11 0FA, United Kingdom; ∥ Institute of Applied Geosciences, Graz University of Technology, Rechbauerstrasse 12, 8010 Graz, Austria; ⊥ Fakultät für Chemie und Pharmazie, Physikalische Chemie, Ludwig-Maximilians-Universität München, Butenandstrasse 5-13, 81377 München, Germany

## Abstract

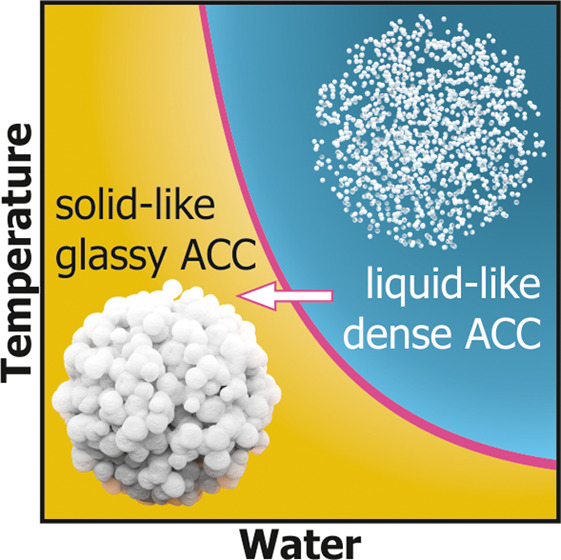

Amorphous carbonates, in their liquid and solid (glassy) forms, have been identified to play important roles in biomineralization, volcanism, and deep element cycling. Anhydrous amorphous calcium and calcium–magnesium carbonate (ACC and ACMC05, respectively) are structural glasses that exhibit a glass transition upon being heated. We report a significant effect of the water content on glass formation. The results yield a parametrization enabling prediction of the stability of their liquid and solid amorphous phases as a function of temperature and water content. These results, obtained through novel fast differential scanning calorimetry, demonstrate that hydrous ACC and ACMC05 do indeed exhibit the behavior of structural glasses and that dehydration of these materials by lyophilization is a route that can be used to isothermally cross the glass transition. This work presents a viable process for a significantly wider range of geo- and biomaterials. Dehydration-controlled formation of glassy ACC therefore constitutes the missing link in the transformation from supersaturated aqueous solutions through an intermediate amorphous glassy state to crystalline CaCO_3_ polymorphs. These results yield direct implications for the mechanistic interpretation of geological processes and biomineralization.

Carbonate melts and glasses are increasingly recognized to play important roles in natural processes. At higher temperatures, the liquid form, carbonate melt, has a low viscosity (i.e., high mobility),^1−3^ compared to most silicate melts, and the potential to accumulate and enrich in economically important elements, such as rare earth elements (REEs).^4,5^ This makes carbonate melt a key phase in deep element cycling and in certain volcanic systems.^6−8^ Amorphous calcium carbonate (ACC) is a transient precursor of a transformation pathway to stable crystalline polymorphs in biomineralization.^9−11^ It has also been reported that biogenic ACC contains up to 5 mol % magnesium (ACMC05).^12^ In addition, ACC has great potential for the development of functional materials such as strong organic/inorganic hybrid materials and as a precursor for defined arranged micro- and nanocrystalline structured materials.^13^ For example, the material has gained attention for its potential utilization in pharmaceutical applications and in the food industry.^14−16^

Amorphous carbonates like ACC (CaCO_3_·*n*H_2_O) and ACMC05 (Ca_0.95_Mg_0.05_CO_3_·*n*H_2_O) are synthesized by lyophilization, which is freezing followed by dehydration through sublimation instead of quenching from a melt. In our previous work,^17,18^ we observed a calorimetric glass transition for anhydrous ACMC05 and anhydrous ACC, which means that an amorphous solid formed by lyophilization is a structural glass, as defined by Angell.^19^ However, the principles of its formation by lyophilization remain poorly understood despite the relevance of amorphous carbonates in a manifold of natural and anthropogenic cases. It has been suggested that different trace and minor components in amorphous carbonates play an important role in the formation and metastability of amorphous carbonates.^20^ Water, in particular, is considered to affect the overall performance of the materials as a precursor for crystalline CaCO_3_ phases.^10,20−25^

Previously, the study of carbonate phases in their supercooled liquid or glass state has been hindered due to their strong tendency to crystallize and dehydrate during thermal analysis at conventional heating rates. Here, we employ fast differential scanning calorimetry (FDSC) with heating rates of up to 2000 °C/s to overcome previous obstacles and to study the glass transition temperature of ACC and ACMC05 as a function of water content.

ACC and ACMC05, as synthesized (see Experimental Methods), are hydrous with 6.89 wt % H_2_O (0.411 mol of H_2_O/mol of ACC) and 8.10 wt % H_2_O (0.486 mol of H_2_O/mol of ACMC05), respectively. Detailed thermal analysis of the hydrous (pristine) amorphous carbonates, as synthesized, is impossible since a large segment of the heat flow curve, and thus, any potential glass transition signal, is masked by a broad endothermic peak associated with dehydration during heating. Hence, we used ACC and ACMC05 dried at 110 °C for 12 h in CO_2_ as the starting material. We found that ACC and ACMC05 contain 3.55 wt % H_2_O (0.204 mol of H_2_O/mol of ACC) and 3.99 wt % H_2_O (0.229 mol of H_2_O/mol of ACMC05), respectively, while FDSC measurements lack the dehydration signal, allowing an unhindered study of the glass transition. Thus, ACC and ACMC05 dried at 110 °C are the most hydrous materials studied in this work. This predried ACC was further dried at 200, 225, and 250 °C to obtain water contents of 1.47, 1.22, and 1.07 wt %, respectively, and predried ACMC05 was further dried at 150, 225, and 250 °C for 6 h in CO_2_ to obtain water contents of 2.74, 1.11, and 0.87 wt %, respectively. Water content analysis was performed by combustion using an elemental analyzer (see Experimental Methods). Although ACC and ACMC05 dried at 250 °C are commonly referred nominally anhydrous, they still contain 1.07 wt % (0.060 mol of H_2_O/mol of ACC) and 0.87 wt % H_2_O (0.048 mol of H_2_O/mol of ACMC05), respectively. Pristine ACC and ACMC05 and ACMC05 dried at 250 °C were studied using transmission electron microscopy.^17,18^ Our observations, e.g., electron diffraction, confirm the preservation of the material’s amorphous structure as indicated by the absence of any signs of crystallinity in electron diffraction patterns. Note that the glass transition temperatures reported in this study are related to the synthesis by lyophilization and to the water content of the amorphous carbonates but not to the drying procedure that was used to obtain samples with different water contents.

We determined the glass transition temperatures for ACC and ACMC05 with water contents from 1.07 to 3.55 wt % and from 0.87 to 3.99 wt %, respectively, at heating rates between 500 and 2000 °C/s up to 450 °C. The temperature of the glass transition increases with heating rate (Figure 1a).

**Figure 1:**
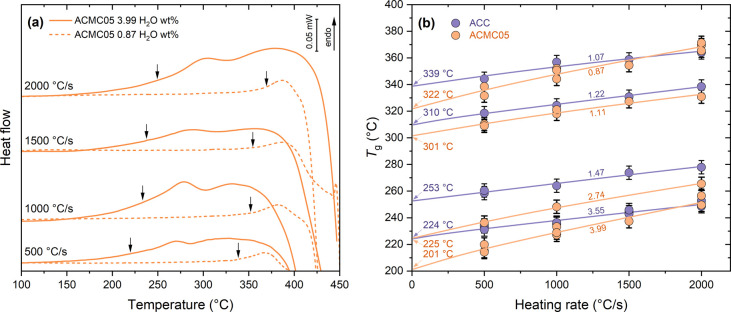
Glass transition temperatures of ACC and ACMC05 with different water contents. (a) Exemplary heat flow curves obtained using FDSC with different heating rates for the most hydrous ACMC05 (solid lines) and the least hydrous ACMC05 (dashed lines). The glass transition temperatures, indicated by black arrows and determined according to Moynihan et al.,^30^ drastically differ between the hydrous and anhydrous samples at similar heating rates. (b) Glass transition temperatures increase with heating rate for ACC (purple) and ACMC05 (yellow). Data for the least hydrous ACC are from ref (18). These trends were fitted by using eq 1 (solid lines) to obtain glass transition temperatures that are corrected for the thermal lag (indicated by arrows). Note the high heating rates of minimum 500 °C/s that are necessary to study the sensitive material.

The amorphous samples always crystallized after crossing the glass transition during heating and subsequent cooling. However, all samples share the same thermal history, which is related to the synthesis. Thus, the glass transition of the prepared sample can be determined, but not the kinetics of the glass transition as a function of the cooling rate, as the glass transition temperature depends on the cooling rate but not on the heating rate.^26^

This also implies that the observed increase in the glass transition temperature with heating rate is due to the thermal inertia of the sample and sensor (thermal lag) τ and must be corrected.^27−29^ The glass transition temperatures for different heating rates were fitted using eq 1(27) (Figure 1b)
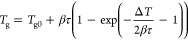
1where *T*_g_ is the measured glass transition temperature, *T*_g0_ is the extrapolated glass transition temperature that is independent of the heating rate and characterizes the initial glass, β is the heating rate, τ is the thermal lag of the sensor and sample, and Δ*T* is the width of the glass transition. *T*_g0_, Δ*T*, and τ are fitted to the experimental values of *T*_g_ and β. All data sets are well fitted by eq 1 with a thermal lag τ between 14 and 34 ms and a Δ*T* between 47 and 75 °C. Hydrous ACC (3.55 wt % H_2_O) and ACMC05 (3.99 wt % H_2_O) yield values of 224 and 201 °C, respectively, the lowest *T*_g0_. The least hydrous ACC (1.07 wt % H_2_O) and ACMC05 (0.87 wt % H_2_O) have glass transition temperatures more than 100 °C higher (at 339 and 322 °C, respectively).

The glass transition temperatures decrease with an increase in water content in both ACC and ACMC05 (Figure 2 and Table 1). A common approach to describe the influence of different components in a system on the glass transition temperature is to use the Gordon–Taylor equation.^31−34^
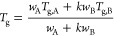
2where *T*_g_ is a glass transition temperature of the mixture, *w*_A_ and *w*_B_ are the weight fractions of both pure end-members (i.e., anhydrous glass and H_2_O, respectively), *T*_g,A_ and *T*_g,B_ are the glass transition temperatures of the two pure substances, and *k* is the Gordon–Taylor parameter characterizing molecular interaction (increases with stronger interaction).^32^ We applied eq 2 to our data, with the *T*_g_ of water being −137 °C determined by Hallbrucker et al.^35^ and Capaccioli and Ngai.^36^*T*_g,A_ and *k* are fitting parameters since the glass transition of anhydrous ACC and ACMC05 is unknown. Values for *T*_gA_ (anhydrous) and *k* are 369 and 13.9 °C for ACC and 333 and 10.3 °C for ACMC05, respectively.

**Figure 2:**
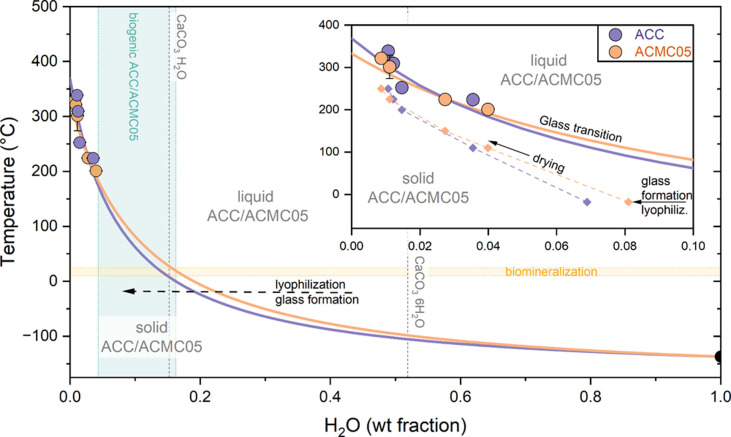
Glass formation in ACC and ACMC05 and implications for biomineralization. Glass transition temperatures for ACC (purple) and ACMC05 (yellow) as a function of water content. The data were fitted using eq 2 (purple line, ACC; yellow line, ACMC05). Dashed gray lines indicate the water content of the hydrous crystalline calcium carbonate polymorphs monohydrocalcite (CaCO_3_·H_2_O) and ikaite (CaCO_3_·6H_2_O). The range of water contents measured in biogenic ACC and ACMC05 is represented by the green vertical band, with the minimum and maximum reported by Radha et al.^10^ and the maximum reported by Koishi et al.^20^ The yellow horizontal band indicates the temperature range associated with biomineralization involving ACC and ACMC05 with limits taken from ref (11) (cave bacteria) and ref (39) (sea urchin). The inset shows a close-up of the *T*_g_ values from this study and water contents attained by drying (diamonds).

**Table 1:** Water Contents of ACC and ACMC05 Used in This Study and Respective Glass Transition Temperatures (*T*_g_) Corrected for the Heating Rate^a^

	H_2_O content		
sample	wt %^c^	mol/mol of carbonate^d^	*T*_g_ (°C)^e^
ACC (pristine)	6.89	0.411	–
ACC-110	3.55	0.204	224
ACC-200	1.47	0.083	253
ACC-225	1.22	0.069	310
ACC-250^b^	1.07	0.060	339
ACMC05 (pristine)	8.10	0.486	–
ACMC05-110	3.99	0.229	201
ACMC05-150	2.74	0.155	225
ACMC05-225	1.11	0.062	301
ACMC05-250	0.87	0.048	322

a Sample names contain the temperature at which the material was dried to obtain the respective water content.

b The *T*_g_ for nominally anhydrous ACC is from ref (18).

c Uncertainty of ±0.05.

d Uncertainty of ±0.005.

e Uncertainty of ±5.

The relationship between the glass transition temperature and water content outlines the stability fields of liquid and solid amorphous phases (Figure 2). From this, it becomes clear that the formation of amorphous carbonates such as ACC and ACMC05, as it occurs for example in biomineralization, can occur via changes in either intensive (decrease in temperature) or extensive properties (removal of water). The observed trend indicates that the synthesis of ACC and ACMC05 glass by lyophilization occurs primarily by dehydration, which increases the glass transition temperature until it can be isothermally crossed. The information about phase stability also helps to clarify why certain crystalline but water-bearing polymorphs, like ikaite (CaCO_3_·6H_2_O), do not exist as amorphous/glass equivalents at least on Earth. Their glass transition temperature is too low to be reached in most natural cases; hence, the crystalline phase is favored at such high water contents.

*A Comparison of Molecular Interactions*. The investigation of the glass transition in ACC and ACMC05 with comparable water contents is motivated by the potential influence of Ca substitution by Mg on the material properties. While ACC is of significant interest in materials science,^13^ the use of ACMC05 is warranted due to the presence of 5 mol % Mg in biogenic ACC,^12^ thereby rendering our findings directly applicable. A Gordon–Taylor analysis of our experimental results reveals that molecular interactions due to the presence of water, expressed as parameter *k*, in ACC (13.9) and ACMC (10.3) are much stronger than those of hydrocarbon/water mixtures (*k* = 1.78–7.30).^32^ This provides a possible explanation for why hydrous amorphous carbonates crystallize relatively slowly, as often previously observed by others,^10,21,23,37,38^ and why such high temperatures are required to dehydrate the material. In addition, the molecular interactions of ACC/H_2_O and ACMC05/H_2_O are stronger by almost a factor of 2 than those in K-Mg-carbonate/H_2_O with a *k* of 7.27,^33^ a unique carbonate composition that can be quenched to glass from a melt. The ACC/water system exhibits stronger molecular interactions compared to those of the ACMC05/water system. This feature provides a possible explanation for the observation that biogenic amorphous carbonates contain impurities of Mg,^10,20^ among others. The glass transition of ACMC05 can be crossed at higher water contents relative to that of ACC at similar temperatures below 200 °C that is relevant for both the synthetic production via lyophilization at a low temperature of −18 °C and the formation of amorphous carbonates in biomineralization (Figure 2). This means that if Mg is incorporated, less entropy is required for isothermal dehydration to form a glass in thermodynamically open systems such as organisms. In other words, small impurities of Mg lead to an entropic advantage.

The vitrification at higher water contents during dehydration at low temperatures also explains the greater water content of ACMC05 synthesized by lyophilization. It should be noted that although FDSC analyses enabled the first exploration of the glass transition in hydrous ACC and ACMC05, the glass transition at water contents similar to those in the biogenic equivalent cannot be directly accessed through calorimetric analysis, even at high heating rates employed in FDSC, making an interpolation necessary.

*Missing Link in the Formation of Solid ACC*. Molecular dynamics simulations of the mechanism of formation of calcium carbonate from supersaturated solutions by Wallace et al.^40^ have shown that hydrated CaCO_3_ clusters are nucleated in a dense liquid phase due to liquid–liquid separation in a supersaturated aqueous medium. The first step in the formation of ACC is the coalescence of nanoscale droplets of this dense ion-rich liquid phase (CaCO_3_·*n*H_2_O). Our study presents the missing link between this dense liquid carbonate phase, which we term liquid ACC, and the structural glass of solid ACC. The findings of Wallace et al. agree with our experimental results because the transient growth and densification are linked to a spatial dehydration (i.e., less water per unit). The glass transition temperature of the dense liquid clusters is thereby increased, and glassy solid ACC can be formed isothermally, which can then act as a precursor for crystalline polymorphs. The same principle likely applies to ACMC05.

The findings presented in this study lead us to conclude that hydrous ACC and ACMC05 are structural glasses formed by lyophilization. Fast differential scanning calorimetry can be applied to determine the water dependence of the glass transition of these sensitive materials. A Gordon–Taylor approach reveals strong molecular interactions in ACC/water and ACMC05/water mixtures compared to hydrocarbon/water mixtures. Finally, dehydration-driven glass formation constitutes the missing link between a dense liquid carbonate phase and solid (glassy) ACC, and the reason for the incorporation of Mg in ACC in biomineralization is the entropic advantage due to the increased water concentration during glass formation by dehydration.

## Experimental Methods

*Synthesis of Amorphous ACC and ACMC05*. Hydrous amorphous calcium carbonate CaCO_3_·*n*H_2_O (ACC) was synthesized following a procedure described in refs (41) and (42). For the synthesis of two ACC solutions, 0.25 M CaCl_2_ and 0.25 M Na_2_CO_3_ were produced from ultrapure deionized water (18.2 MΩ cm^–1^), and CaCl_2_·2H_2_O, MgCl_2_·6H_2_O, and Na_2_CO_3_ were obtained from Carl Roth chemicals. The solutions were cooled in a refrigerator at 10 ± 1 °C for a minimum of 4 h. ACC was precipitated by mixing 80 mL of CaCl_2_ with 80 mL of a Na_2_CO_3_ solution. Precipitated ACC was isolated using a 0.2 μm cellulose filter and a suction filtration unit followed by drying for 12 h in a Virtis Benchtop 3L freeze-dryer.

Hydrous amorphous calcium–magnesium carbonate Ca_0.95_Mg_0.05_CO_3_·*n*H_2_O (ACMC05) was synthesized using the same procedure but using a 0.25 M (Ca,Mg)Cl_2_ solution instead of the CaCl_2_ solution. Purgstaller et al.^42^ found that the resulting Mg in ACMC05 follows Mg_ACMC_ = – 21.03243[1 – exp(0.0175 × Mg_stock_)], where  (in percent). Thus, to obtain solid ACMC05 with 5 mol % Mg, a 12 mol % stock solution was used.

Hydrous ACC and ACMC05 were held at 110 °C in air for 3 weeks to prevent adsorption of water from the atmosphere, possible rehydration, and crystallization. The so-produced ACC-110 and ACMC05-110 were still found to be amorphous and hydrous. The material was kept in a desiccator with silica gel (3% relative humidity) throughout this study. ACC-110 and ACMC05-110 were used as the starting material for all analyses presented in this study.

*Elemental Analysis*. Water contents of the carbonate glasses were analyzed with a ThermoScientific FlashSmart elemental analyzer that operates on a modified Dumas method. First, 2–5 mg of amorphous carbonate was enclosed in tin containers and combusted in high-purity oxygen. Helium acted as the carrier gas for the transport to an adjacent gas chromatograph. Hydrogen was detected by its thermal conductivity. From this weight percent, the mass of H_2_O was calculated relative to the initial sample mass.

The elemental analyzer was calibrated using a BBOT standard and checked with secondary standards before and after the measurement of three replicates of each sample.

*Fast Differential Scanning Calorimetry (FDSC)*. DSC measurements of ACC and ACMC05 were performed using a fast differential scanning calorimeter (Mettler-Toledo Flash DSC 2+). The FDSC instrument was equipped with a UFS1 chip sensor, as described by van Herwaarden et al.^43^ This MEMS technology-based sensor provides heating rates of up to 40 000 °C/s and maximum cooling rates of 4000 °C. In this study, we use a maximum heating and cooling rate of 2000 °C/s to reduce the effects of thermal inertia due to the relatively large contact resistance between the sample and the sensor. A thin layer of silicon oil (AK 500.000 Wacker) was applied to the sensor surface before samples were placed on the sensor to homogenize the thermal contact and to mitigate the probability of jumping sample particles during measurements. A homogeneous oil film was formed by a heating–cooling cycle (six cycles at 2000 °C/s to 300 °C) after a small oil drop was placed on the sensor and before the sample was positioned on the sensor.

High-angle annular dark-field scanning transmission electron microscopy showed that the micrometer-sized grains of ACC and ACMC05 consisted of agglomerates of nanosized colloids.^17,18^ Samples for FDSC analysis were picked from these powders using fine-tipped hair and deposited directly onto the lubricated sensor. Ideally, samples were tabularly shaped agglomerates to enhance the thermal dispersion throughout the samples upon heating and cooling cycles. These were placed on the sensor and carefully pressed to improve the contact. The sensor was flushed with CO_2_ at a flow rate of 30 mL/min to inhibit the thermal decomposition of the carbonates.

To prevent the evaporation of weakly bound water that overlaps with the glass transitions in the DSC curve, the sample must be dried before the measurements. Each sample was dried on the sensor isothermally at 110, 150, 200, 225, and 250 °C for 10 min at each temperature. After this procedure, no weakly bound water evaporated. The glass transition was measured during subsequent heating between 30 and 500 °C. After cooling at matching rates, the heating segment was repeated. Such measurements were performed at 500, 1000, 1500, and 2000 °C/s by using fresh samples for each scanning rate. Similar sample sizes were chosen for all measurements, while slightly larger samples were used at low heating rates.

The original goal was to heat the sample shortly above the glass transition and cool it rapidly to prevent crystallization. In the frame of the usable cooling rates, the prevention of crystallization during cooling was not possible. Consequently, only in the first heating run could the glass transition be detected. The second heating run measured the heat flow of the crystalline material. This curve was used as a baseline.

The glass transition temperature was defined as the limiting fictive temperature as proposed by Richardson and Savill^44^ and Moynihan et al.^30^ This is a thermodynamically defined characteristic temperature describing the configurational entropy of the glass. In the used evaluation software of Mettler-Toledo, this glass transition temperature is named the “glass transition temperature according to Richardson”.

## Data Availability

The data produced and analyzed during this study are available from the corresponding author upon reasonable request.

## Supporting Information Available

Description of the FDSC analysis data for heat flow curves shown in Figure 1a (PDF)
FDSC heat flow curves that were used for Figure 1a (ZIP)
Transparent Peer Review report available (PDF)
