# Supplementary material for: Dehydration-Driven Glass Formation in Aqueous Carbonates
Source: J Phys Chem Lett. 2025 May 7;16(19):4773–9. doi: 10.1021/acs.jpclett.5c00551 (PMC12086832; doi:10.1021/acs.jpclett.5c00551)
Supplement: Supplementary file 1 — jz5c00551_si_001.pdf [file jz5c00551_si_001.pdf]

Supporting Information for

Dehydration-driven glass formation in aqueous carbonates

Thilo Bissbort[1], Kai-Uwe Hess[1], Daniel Weidendorfer[1], Elena V. Sturm[1], Jürgen E. K. Schawe[2], Martin Wilding[3], Bettina Purgstaller[4], Katja E. Goetschl[4], Sebastian Sturm[5], Knut Müller-Caspary[5], Wolfgang Schmahl[1], Erika Griesshaber[1], Martin Dietzel[4], Donald B. Dingwell[1]*

[1]Earth and Environmental Sciences, Ludwig-Maximilians-Universität München, Theresienstraße 41/III, 80333 München, Germany

[2]Laboratory of Metal Physics and Technology, Department of Materials, ETH Zurich, 8093 Zurich, Switzerland

[3]UK Catalysis Hub, Research Complex at Harwell, Rutherford Appleton Laboratory, Harwell Campus, Oxfordshire OX11 0FA, United Kingdom

[4]Institute of Applied Geosciences, Graz University of Technology, Rechbauerstrasse 12, 8010 Graz, Austria

[5]Fakultät für Chemie und Pharmazie, Physikalische Chemie, Ludwig-Maximilians-Universität München, Butenandstr. 5-13, 81377, München, Germany

* Corresponding author

(1) FDSC analysis data for heat flow curves shown in Fig 1a

FDSC heat flow curves which were used for Fig 1a have been made accessible as .txt files and can be downloaded from https://doi.org/10.5281/zenodo.12665436.
