# Supplementary material for: Dehydration-Driven Glass Formation in Aqueous Carbonates
Source: J Phys Chem Lett. 2025 May 7;16(19):4773–9. doi: 10.1021/acs.jpclett.5c00551 (PMC12086832; doi:10.1021/acs.jpclett.5c00551)
Supplement: Supplementary file 3 — jz5c00551_si_003.pdf [file jz5c00551_si_003.pdf]

jz-2025-00551f.R1

Name: Peer Review Information for "Dehydration-driven glass formation in aqueous carbonates"

First Round of Reviewer Comments

Reviewer: 1

Comments to the Author

The manuscript entitled “Dehydration-driven glass formation in aqueous carbonates” by Bissbort et al. reports an interesting application of fast scanning calorimetry. By using heating rates around 1000 K/s, evaporation of bound water was successfully avoided, and the glass transition in dependence on water content became accessible. The strategy of the experiments is well described, and the results obtained by extrapolating the Gordon-Taylor equation into the relevant concentration range for biomineralization provides new insights into the relevant processes. The manuscript is well-prepared and can be published as is.

The present reviewer cannot judge the relevance of the obtained results for biomineralization or other processes mentioned in the manuscript.

Author's Response to Peer Review Comments:

Dear Editor,

We sincerely appreciate your editorial handling of our manuscript and are pleased to receive such a positive review. We are submitting our revised manuscript, with modifications limited to the formatting changes requested in the associated email. A summary of these minor revisions is provided below.

1. The affiliation "[2]Mettler-Toledo GmbH, Heuwinkelstrasse 3, CH-8603, Nänikon, Switzerland" was deleted from the title page. The numbering changed accordingly.

2. The corresponding author is now indicated with an asterisk in the author list on the manuscript title page.

3. The TOC graphic is now fitted into an area of 8.21 x 4.45 cm (width x height) in the word document.

4. The page numbers of reference 18 were added to the associated entry in the reference list.

5. The entry for reference 19 was completed in the reference list and is now: “Angell, C. A. Glass Transition. In Encyclopedia of Materials: Science and Technology; Elsevier, 2004, pp. 1–11.”

6. The supplementary material mentioned in the “Associated content” section is accessible by following the link given in this section. However, we have now uploaded these files (.zip folder) with our revision.

7. The following sentence was added to the acknowledgement: “The authors express their gratitude to Juan Bisquert for his editorial handling and to the anonymous reviewer for their valuable feedback.”
